# Spatial and temporal distribution of *Culicoides* species in the New England region of New South Wales, Australia between 1990 and 2018

**DOI:** 10.1371/journal.pone.0249468

**Published:** 2021-04-05

**Authors:** Biniam T. Lakew, Adrian H. Nicholas, Stephen W. Walkden-Brown

**Affiliations:** 1 Animal Science, School of Environmental and Rural Science, University of New England, Armidale, New South Wales, Australia; 2 College of Veterinary Medicine, Haramaya University, Dire Dawa, Ethiopia; 3 Department of Regional New South Wales, Central Coast Primary Industries Centre, Ourimbah, New South Wales, Australia; University of Waikato, NEW ZEALAND

## Abstract

*Culicoides* are one of the smallest hematophagous flies measuring 1–5 mm in size with only females seeking blood for egg development. The present study investigated spatio-temporal distribution of *Culicoides* species trapped between 1990 and 2018 at 13 sites in the New England region of NSW, Australia using automated light traps. Trapping locations were divided into three subregions (tablelands, slopes and plains). Nineteen *Culicoides* species were identified. *Culicoides marksi* and *C*. *austropalpalis* were the most abundant and widespread species. *Culicoides brevitarsis*, the principal vector of livestock diseases in New South Wales comprised 2.9% of the total catch and was detected in 12 of the 13 locations in the study. Abundance as determined by Log_10_ *Culicoides* count per trapping event for the eight most abundant species did not vary significantly with season but trended towards higher counts in summer for *C*. *marksi* (P = 0.09) and *C*. *austropalpalis* (P = 0.05). Significant geographic variation in abundance was observed for *C*. *marksi*, *C*. *austropalpalis* and *C*. *dycei* with counts decreasing with increasing altitude from the plains to the slopes and tablelands. *Culicoides victoriae* exhibited the reverse trend in abundance (P = 0.08). Greater abundance during the warmer seasons and at lower altitudes for *C*. *marksi* and *C*. *austropalpalis* was indicative of temperature and rainfall dependence in this region with moderate summer dominance in rainfall. The Shannon-Wiener diversity index of species was higher on the tablelands (H = 1.59) than the slopes (H = 1.33) and plains (H = 1.08) with evenness indices of 0.62, 0.46 and 0.39 respectively. *Culicoides* species on the tablelands were more diverse than on the slopes and plains where *C*. *marksi* and *C*. *austropalpalis* dominated. The temporal and spatial variation in abundance, diversity and evenness of species reported in this diverse region of Australia provides additional insight into *Culicoides* as pests and disease vectors and may contribute to future modelling studies.

## Introduction

Biting midges, *Culicoides* Latreille (Diptera: Ceratopogonidae), are one of the smallest hematophagous flies measuring 1–5 mm in size [1–3]. The family is divided into 6,206 recognized extant valid species arranged in three subfamilies and 112 genera [4, 5]. The presence of *Culicoides*, *Leptoconops* Skuse, *Austroconops* Wirth and Lee, and the sub-genera *Lasiohelea* Kieffer which feed on blood of vertebrates has been reported in Australia [6–8]. In the genus *Culicoides*, only females seek blood for egg development [1]. Borkent and Dominiak [5] created a list of 1,347 *Culicoides* species occurring from tidal areas to the highest mountain peaks of up to 4,651 meters and on all continents with exception of the extreme polar regions and New Zealand [1, 9, 10].

Several species of *Culicoides* are considered as vectors for a wide array of viruses, bacteria and nematodes affecting a wide range of domestic and wild animals [11]. In Australia, Barmah Forest virus (Alphavirus), Eubenangee and Warrego viruses (Orbivirus) were isolated from *C*. *marksi* Lee and Reye, and Wallal virus (Orbivirus) from *C*. *dycei* Lee and Reye [12]. However, bluetongue and Akabane viruses are of most importance to the livestock industry with the former having a significant impact on global sheep and cattle trade [13–15]. In Australia, proven vectors of bluetongue virus are *C*. *actoni* Smith, *C*. *fulvus* Sen and Das Gupta and *C*. *brevitarsis* Kieffer, while *C*. *dumdumi* Sen and Das Gupta, *C*. *oxystoma* Kieffer, *C*. *peregrinus* Kieffer and *C*. *wadai* Kitaoka are regarded as probable vectors [16, 17]. Surveillance on the spatial and temporal distribution of these vectors is continuously monitored by the National Arbovirus Monitoring Program (NAMP) [18].

*Culicoides* can fly up to 151 meters above the ground [19, 20] with flight activity in search of hosts limited to less than 2 kms [21]. However, they can be transported passively as aerial plankton over greater distances on a prevailing wind [14, 22]. There are reports of long-range introduction of *Culicoides* from Indonesia, Timor-Leste and Papua New Guinea into northern Australia especially during the monsoon season [9, 23]. The Australian continent is a large landmass, which is mostly flat favouring the potential for long-distance spread of *Culicoides* under suitable conditions. Continuous monitoring of *Culicoides* arbovirus vector species is important for establishing and maintaining protocols for the Australian livestock export industry.

Globally, the distribution of vectors and vector-borne diseases of livestock and humans is increasing at an alarming rate [24] with distribution of vectors dependent on changes related to climate, environment, an increase in host mobility, unplanned urbanization and agricultural intensification [24, 25]. In Australia, Standfast and Muller [26] predicted a “Greenhouse Effect” on the distribution of *C*. *wadai* with a 3°C rise in minimum temperature and 20% increase in summer rainfall leading to incursion of this species into new areas. More specifically, changes in distribution are associated with the seasonal movement of vectors resulting from changes in temperature, precipitation, soil moisture and host availability [27].

The Australian *Culicoides* fauna is extensive and diverse with species presence and distribution determined by geography, weather and breeding habitat preferences [28–30]. For example, *C*. *brevitarsis* and *C*. *wadai* breed exclusively in bovine dung pats [31]. Dyce et al. [10] provided a comprehensive list of Australian *Culicoides* species of which 49 species are present in New South Wales (NSW). This paper reports *Culicoides* species composition, abundance and spatio-temporal distribution based on trapping results from the New England region of NSW between 1990 and 2018.

## Materials and methods

### Description of the New England region

**Fig 1:**
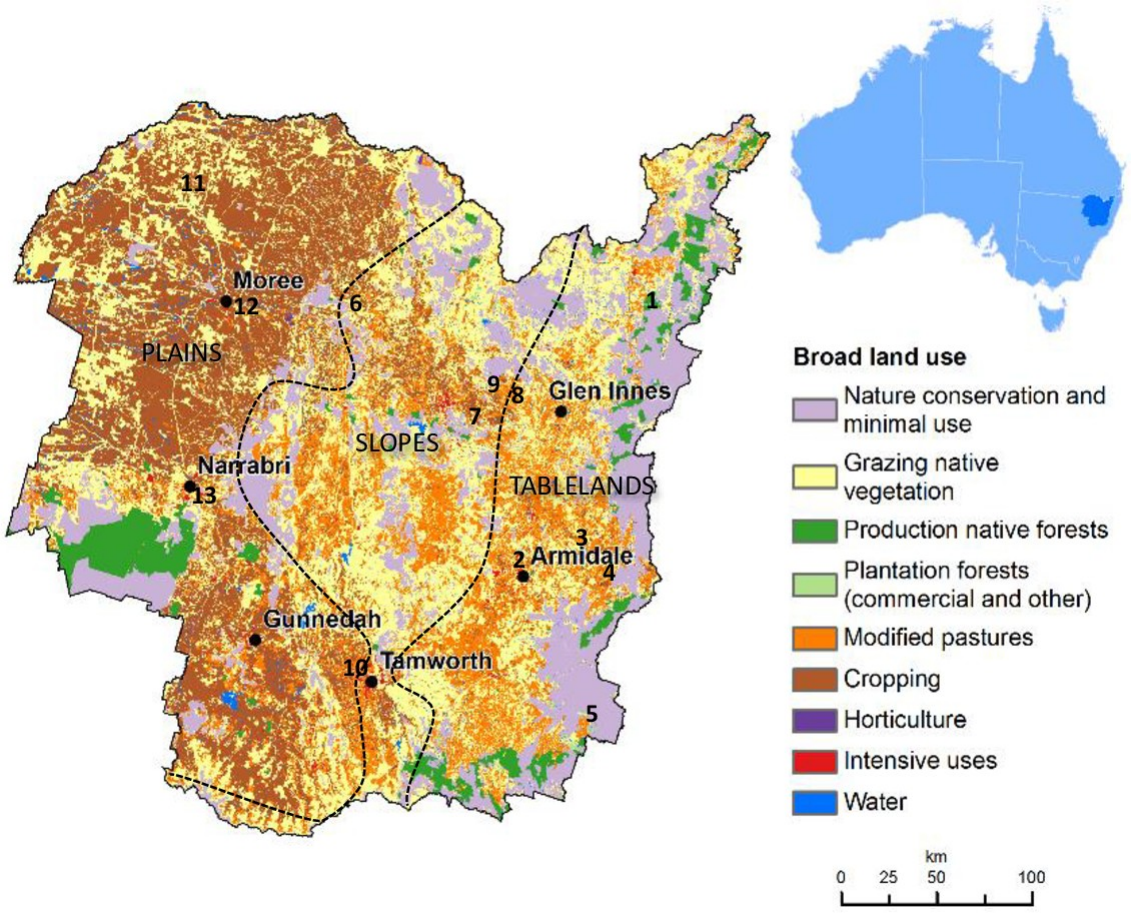
Map on the land use practices in the New England region of NSW, Australia [33] with the approximate boundaries of the tablelands, slopes and plains areas and location of the sampling sites (numbered) superimposed. Reprinted from [Catchment Scale Land Use of Australia] under a CC BY license, with permission from [Australian Bureau of Agricultural and Resource Economics and Sciences, ABARES], original copyright [2019].

The New England region of New South Wales is located in the north of the state and west of the Great Dividing Range (GDR), including the tableland areas around Armidale and north to the Queensland border. It covers a total area of 99,100 km^2^ or 12% of the state with agricultural and conservation lands comprising 79,400 km^2^ (80%) and 14,400 km^2^ (15%) respectively [32]. The most common land use in the area is grazing upon modified pastures which occupies 39,200 km^2^ (40%) [33]. The New England region generally declines in altitude from east to west and can be broadly categorized into Tablelands (800–1400 m altitude), Slopes (400–800 m) and Plains (<400 m) subregions [34, 35] with varied land use as shown in Fig 1. Grazing areas are dominated by sheep and cattle, and various common wildlife species [36].

### Trapping locations

As part of the NAMP monitoring of *Culicoide*s-borne arboviruses of ruminant livestock in Australia [37], *Culicoides* trapped from 34 sites on cattle properties at thirteen locations in the New England region between 1990 and 2018 were used in this study (Table 1). Trap sites were occasionally redeployed to nearby cattle property sites within locations following destocking events. For the location Mungindi, there were few trapping events and the data sets generated were merged with data from the nearby site, Boomi. Details of trapping locations used with their basic climatic conditions are provided in Table 1.

**Table 1:** Details on the locations and climate data during *Culicoides* trappings from 1990 to 2018 in the New England region of NSW, Australia.

Locations	Alt(m)	Subregions	Mean annual Rainfall (mm)	Mean minimum and maximum temperature (°C)								Sites^a^
				Spring		Summer		Autumn		Winter		
				T_min_	T_max_	T_min_	T_max_	T_min_	T_max_	T_min_	T_max_	
1. Tenterfield	850	Tablelands	784.2	7.6	22.5	14.0	26.3	8.3	19.8	1.3	15.9	2
2. Armidale	980	Tablelands	749.1	7.4	20.3	12.8	25.4	7.5	18.7	1.8	12.9	2
3. Wollomombi	964	Tablelands	735.6	7.4	20.3	12.8	25.4	7.5	18.7	1.8	12.9	1
4. Jeogla	964	Tablelands	875.7	7.4	20.3	12.8	25.4	7.5	18.7	1.8	12.9	1
5. Yarrowich	940	Tablelands	1045.8	11.8	25.7	17.3	28.8	12.7	22.5	6.5	19.5	2
6. Wallangra	550	Slopes	725.9	10.2	26.1	17.0	31.2	10.3	23.8	2.5	18.7	4
7. Inverell	582	Slopes	791.3	7.9	24.6	14.6	29.9	7.4	23.5	0.5	17.4	3
8. Swanvale	780	Slopes	779.8	7.2	20.7	12.9	25.2	7.9	18.8	1.3	13.7	1
9. Brodies Plains	620	Slopes	786.4	7.9	24.6	14.6	29.9	7.4	23.5	0.5	17.4	1
10. Tamworth	404	Slopes	631.9	9.6	25.5	16.7	31.6	10.1	23.4	2.8	17.3	7
11. Mungindi-Boomi	184	Plains	583.9	13.9	28.7	20.7	35.0	12.9	26.5	5.8	20.6	6
12. Moree	212	Plains	568.3	12.7	27.8	19.4	33.3	12.2	25.9	5.2	19.2	1
13. Narrabri	212	Plains	618.9	12.1	27.8	19.3	33.7	11.4	25.3	4.8	18.8	3

^a^The number of times trapping sites were changed to different farms.

Alt: Altitude; T_min_: Mean Minimum Temperature; T_max_: Mean Maximum Temperature. (Details for the climate data between 1990 and 2018 sourced from archival records at: www.bom.gov.au).

### Trap types and deployment

Most *Culicoides* species are crepuscular and can be sampled using light traps [38, 39]. Initially, the NAMP used CDC-style light traps fitted with incandescent globes (1990 to 2005) as described previously [38]; however, in 2005 the traps were retrofitted with three green light-emitting diodes (LED) as these were proven efficient in collecting arbovirus vector species [40]. The lamp design was further upgraded in 2016 with eight 5 mm, 525 nm LEDs with an intensity of 21000 mcd (Millicandela). These are battery powered and a sensor automatically switches the light and fan on at dusk and off at dawn. The fan draws *Culicoides* attracted to the light source down into the capture bottle containing 100 ml of 80% ethanol.

The traps were deployed for approximately two nights per month in the week of the new moon. This prevented insects from being distracted from the light traps by moonlight. Similarly, traps were not deployed in the vicinity of buildings where light may also distract the *Culicoides* from the trap. Most trap deployments were made in the spring, summer and autumn months.

### Identification of *Culicoides* species

Trapped *Culicoides* were separated from by catch using morphological features, preserved in 80% ethanol and held in sealed containers away from light to prevent UV or sunlight damage. Though morphological identification of cryptic species is not reliable as mentioned previously [41], the *Culicoides* were identified to species using their wing patterns by entomologists at the Department of Regional NSW (DRNSW), Central Coast Primary Industries Centre in accordance with the pictorial wing atlas [10].

### Data management and analysis

No trapping was done in 1993 and no catches made in 1994, so these years were excluded from the analysis. Similarly, the very small number of trapping events in winter were excluded from the dataset. The annual trapping period went from early spring (September) until the end of autumn (May) resulting in a total of 26 trapping years. The MS-Excel spreadsheets data from 13 trapping locations were merged and the final dataset comprised records of 4421 trapping events. However, only eight *Culicoides* species representing 99.2% of the total count were considered for the analysis.

The data were analysed using JMPv.14 statistical software (SAS Institute Inc., Cary, NC, USA). *Culicoides* per trapping event (Count) were Log_10_ transformed [y = Log_10_ (x+1)] to meet the assumptions of analysis of variance. Analysis of counts of each *Culicoides* species for the repeated measure effects: Years (trapping years 1990/91-2017/18), Seasons [spring (September to November), summer (December to February), autumn (March to May)] and Subregions (tablelands, slopes, plains) and their interactions were fitted as fixed effects in a mixed REML (restricted maximum likelihood model) with trapping location fitted as a random variable. The effect of trap light changes; incandescent globe (1990/91-2004/05), Change 1 (3 green LED; 2005/06-2015/16) and Change 2 (8 green LED; 2016/17-2017/18) on *Culicoides* counts were fitted as repeated measures model with location as a random effect and analysed using one-way ANOVA. The significance of differences between means within an effect was tested using Tukey’s HSD test. To test differences in binomial data (e.g. success or failure of trapping events), contingency table analysis was used with the Pearson Chi-Square test. A significance value of P<0.05 is used throughout with count data presented as least squares means and standard error (LSM ± SE).

The Shannon-Wiener diversity index (*H*) was used to determine diversity of *Culicoides* species across the three subregions using the formula below:

$$H=-\sum_{i=1}^{S}P_{i}lnP_{i}$$

Where the proportion of species (*i*) relative to the total number of species (*Pi*) was calculated and multiplied by the natural logarithm of this proportion (*lnPi*). The result is summed across species and multiplied by -1 [42]. The evenness index (*E*) was used to assess the homogeneity or pattern of distribution of species in relation to other species.

E=H/lnS

Where H is the number derived from the Shannon-Wiener diversity index and S is the total number of species. This index varies between 0 and 1, with 1 showing complete evenness.

## Results

### Species composition and abundance

A total of 152,629 *Culicoides* were trapped between 1990/91 and 2017/18 from thirteen locations in the New England region of NSW. From total green LED (n = 2320) and incandescent (n = 2101) light trap deployments, 88.8% and 81.0% respectively resulted in successful catches. More than a 10-fold increase in abundance and a rise in species diversity from six to ten was observed when the green LED was introduced in 2005/06 (S1 Table). Overall, a total of nineteen species were identified with *C*. *marksi* (38.6%), *C*. *austropalpalis* Lee and Reye (36.7%), *C*. *victoriae* Macfie, (9.6%), *C*. *dycei* (6.6%), *C*. *bundyensis* Lee and Reye (2.9%), *C*. *brevitarsis* (2.9%) and *C*. *nattaiensis* Lee and Reye (1.1%) being the most abundant species. *Culicoides shermani* Causey, *C*. *parvimaculatus* Lee and Reye and *C*. *moreensis* Lee and Reye were the least common species (Table 2).

**Table 2:** Abundance of *Culicoides* species trapped from the New England region of NSW, Australia between 1990 and 2018 showing years, seasons and locations of trapping.

S/N	*Culicoides* spp.	No. of times detected	No. of midges trapped	Mean F/D^a^	Locations detected^b^	No. of years trapped (n)	Detected in trap types	Seasons detected^c^
1	*C*. *marksi*	638	58932	92	All (13)	26	Both	3 (Sp, Su &A)
2	*C*. *austropalpalis*	814	56029	69	All	26	Both	3 (Sp, Su &A)
3	*C*. *victoriae*	807	14710	18	All	26	Both	3 (Sp, Su &A)
4	*C*. *dycei*	366	10093	28	12 (not 4)	25	Both	3 (Sp, Su &A)
5	*C*. *bundyensis*	368	4392	12	All	24	Both	3 (Sp, Su &A)
6	*C*. *brevitarsis*	238	4361	18	12 (not 9)	22	Both	3 (Sp, Su &A)
7	*C*. *nattaiensis*	166	1734	10	All	25	Both	3 (Sp, Su &A)
8	*C*. *bunrooensis*	186	1097	6	11 (not 3 & 4) & 3	25	Both	3 (Sp, Su &A)
9	*C*. *fulbrighti*	72	925	13	2, 7, 4, 1, 6, 3 & 5	15 (>1999/2000)	Both	3 (Sp, Su &A)
10	*C*. (Ornatus Gp sp) **#**8	9	144	16	12, 11, 13, 10 & 6	3 (>2015/16)	LED	2 (Su &A)
11	*C*. *marginalis*	20	50	3	7, 12, 11, 8, 10 & 6	6 (>2011/12)	Both	2 (Su &A)
12	*C*. *rabauli*	20	39	2	2, 7, 13, 10, 6, 3 & 5	9 (>2004/05)	Both	2 (Su &A)
13	*C*. *williwilli*	14	38	3	2, 9, 7, 11, 10, 6, 3, & 5	7 (infrequent)	Both	2 (Su &A)
14	*C*. *zentae*	11	36	3	9, 7, 11, 8, & 10	2 (1990/91&1991/92)	Incandescent	3 (Sp, Su &A)
15	*C*. *sigmoidus*	18	28	2	2, 7, 1, 3, & 5	12 (>1999/2000)	Both	3 (Sp, Su &A)
16	*C*. *loughnani* Edwards	9	13	1	7, 12, 10 & 6	6 (>2010/11)	LED	2 (Su &A)
17	*C*. *shermani*	4	5	1	7, 11 & 13	3 (infrequent)	Incandescent	2 (Su &A)
18	*C*. *parvimaculatus*	2	2	1	7 & 11	1 (1991/92)	Incandescent	1 (Su)
19	*C*. *moreensis*	1	1	1	3	1 (2017/18)	LED	1 (A)

^a^Mean F/D: Mean number of flies per detection.

^b^Numbers correspond to respective trapping locations as per Table 1.

^c^Season: Sp-spring, Su-summer and A- autumn.

### Temporal and spatial distribution of *Culicoides* species

The highest number of detections, i.e., the success of trapping at least one *Culicoides* specimen, was in the trapping year 2017/18 (97.8%) and the lowest in 1996/97 (65.9%). *Culicoides marksi*, *C*. *austropalpalis* and *C*. *victoriae* were detected in all sampling years whereas *C*. *parvimaculatus* and *C*. *moreensis* were only detected in a single year (Table 2). *Culicoides* (Ornatus Gp sp) **#**8 was detected annually from 2015/16. An increase in the relative abundance of *Culicoides* species was observed when the green LED trap was introduced in 2005/06 (Change 1) and when the LED lamp design was upgraded in 2016/17 (Change 2). However, catches of *C*. *nattaiensis* were significantly higher (<0.0001) prior to LED trap introduction with details of abundances for the remainder species shown below (Table 3). Overall, of the 19 species identified, 13 (68%) were detected in both trap types whereas 3 (16%) were trapped with incandescent (CDC-style) lights only and 3 with green LED traps only as shown above in Table 2.

**Table 3:** Variation in temporal abundance of the eight most abundant *Culicoides* spp trapped in the New England region of NSW, Australia between 1990/91 and 2017/18 due to trap type improvements.

*Culicoides* spp.	Log_10_ *Culicoides* count/trapping event (Mean ± SE)			P-value
	CDC-style	green LED^a^	LED-improved^b^	
*C*. *marksi*	0.94 ± 0.04^b^	1.05 ± 0.05^b^	1.66 ± 0.09^a^	<0.0001
*C*. *austropalpalis*	0.92 ± 0.03^c^	1.06 ± 0.04^b^	1.76 ± 0.08^a^	<0.0001
*C*. *victoriae*	0.64 ± 0.03^c^	0.86 ± 0.03^b^	1.33 ± 0.06^a^	<0.0001
*C*. *dycei*	0.84 ± 0.04^b^	0.91± 0.05^b^	1.13 ± 0.09^a^	0.01
*C*. *bundyensis*	0.64 ± 0.04^b^	0.71 ± 0.03^b^	0.99 ± 0.07^a^	<0.0001
*C*. *brevitarsis*	0.65 ± 0.06^a^	0.75 ± 0.04^a^	0.80 ± 0.10^a^	0.28
*C*. *nattaiensis*	0.81 ± 0.05^a^	0.47 ± 0.06^b^	0.67 ± 0.09^a^^b^	<0.0001
*C*. *bunrooensis*	0.51 ± 0.04^b^	0.54 ± 0.04^b^	0.89 ± 0.07^a^	<0.0001

^a^Represents mean *Culicoides* counts between 2005/06 and 2015/16 (Change 1).

^b^Represents mean *Culicoides* count when the LED lamp design was modified since 2016/17 (Change 2). Means in rows with the same letter or case are not significantly different (P<0.05).

Of the 4421 trapping events (663 in spring, 2086 in summer and 1672 in autumn), 85.1% resulted in successful *Culicoides* catches. Analysis of the effect of season on the mean *Culicoides* count (Log_10_ count/trapping event) revealed no significant difference with details of seasonal variations in catches of the eight most abundant species shown below (Table 4). However, there was a non-significant trend towards lower catches of *C*. *marksi* (P = 0.09) and *C*. *austropalpalis* (P = 0.05) in spring.

**Table 4:** Variation on *Culicoides* species abundance across seasons and subregions trapped in the New England region of NSW, Australia between 1990 and 2018.

*Culicoides* spp.	Log_10_ count by season (LSM ± SE)			P-value	Log_10_ count by subregions (LSM ± SE)			P-value
	Spring	Summer	Autumn		Tablelands	Slopes	Plains	
*C*. *marksi*	0.78 ± 0.09^a^	0.94 ± 0.07^a^	0.85 ± 0.07^a^	0.09	0.38 ± 0.11^b^	0.89 ± 0.11^a^	1.30 ± 0.11^a^	0.002
*C*. *austropalpalis*	0.86 ± 0.10^a^	1.01 ± 0.08^a^	0.93 ± 0.09^a^	0.05	0.48 ± 0.14^b^	1.04 ± 0.14^a^	1.28 ± 0.16^a^	0.009
*C*. *victoriae*	0.72 ± 0.09^a^	0.71 ± 0.06^a^	0.69 ± 0.07^a^	0.95	0.85 ± 0.08^a^	0.79 ± 0.09^a^	0.48 ± 0.13^a^	0.08
*C*. *dycei*	0.71 ± 0.15^a^	0.69 ± 0.09^a^	0.58 ± 0.09^a^	0.40	0.39 ± 0.18^b^	0.61 ± 0.12^ab^	0.98 ± 0.12^a^	0.03
*C*. *bundyensis*	0.56 ± 0.11^a^	0.67 ± 0.10^a^	0.66 ± 0.09^a^	0.66	0.67 ± 0.09^a^	0.65 ± 0.10^a^	0.57 ± 0.16^a^	0.85
*C*. *brevitarsis*	0.50 ± 0.50^a^	0.60 ± 0.09^a^	0.62 ± 0.08^a^	0.92	0.66 ± 0.18^a^	0.49 ± 0.20^a^	0.56 ± 0.25^a^	0.45
*C*. *nattaiensis*	0.63 ± 0.11^a^	0.61 ± 0.07^a^	0.52 ± 0.08^a^	0.41	0.57 ± 0.12^a^	0.42 ± 0.11^a^	0.78 ± 0.13^a^	0.21
*C*. *bunrooensis*	0.67 ± 0.12^a^	0.60 ± 0.07^a^	0.66 ± 0.08^a^	0.62	0.75 ± 0.14^a^	0.55 ± 0.09^a^	0.63 ± 0.10^a^	0.47

Means in rows with the same letter or case are not significantly different (P<0.05).

**Fig 2:**
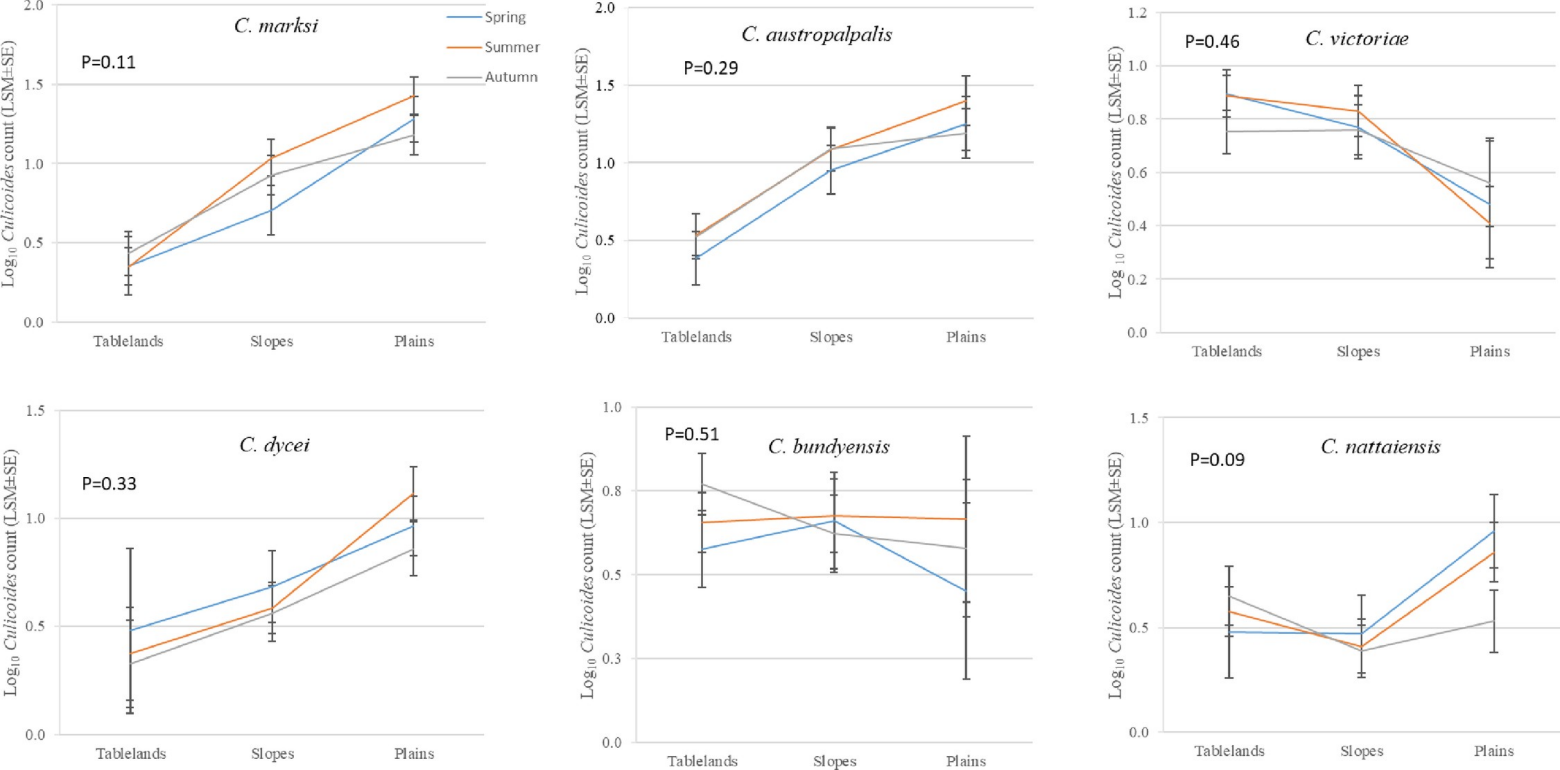
Effect of season and subregion on counts per trapping event (Log_10_, LSM ± SE) of the most abundant *Culicoides* species trapped in the New England region of NSW, Australia between 1990 and 2018.

The most widespread species were *C*. *marksi*, *C*. *austropalpalis*, *C*. *bundyensis*, *C*. *nattaiensis* and *C*. *victoriae* (present in thirteen locations) followed by *C*. *brevitarsis* and *C*. *dycei* (twelve locations) whereas *C*. *moreensis* was the least dispersed species as shown in Table 2. There were significant subregional differences in abundance for *C*. *marksi* (P = 0.002), *C*. *austropalpalis* (P = 0.009) and *C*. *dycei* (P = 0.03) all showing a decline in abundance with increasing elevation from the plains to the slopes and tablelands (Table 4). *Culicoides victoriae* on the other hand exhibited a non-significant trend (P = 0.08) in the opposite direction (Table 4). There were no significant interactions between the effects of season and subregion for the most abundant *Culicoides* species (Fig 2).

### Shannon-Wiener diversity and evenness indices

The tablelands, slopes and plains have distinct geographical characteristics and therefore were treated as discrete habitats. The index for diversity of species on the tablelands (H = 1.59) was higher than the slopes (H = 1.33) and plains (H = 1.08) (Table 5). The same trend was observed for evenness index with values for the tablelands, slopes and plains being 0.62, 0.46 and 0.39 respectively. These findings clearly indicate that distribution of species on the tablelands was more diverse and even than on the slopes and plains where *C*. *marksi* and *C*. *austropalpalis* dominated.

**Table 5:** Shannon-Wiener diversity and evenness indices of *Culicoides* species in New England subregions of NSW, Australia.

*Culicoides* species	Total catch in subregions			
	Tablelands (5)^a^	Slopes (5)	Plains (3)	Total
*C*. *austropalpalis*	4410	18210	33409	56029
*C*. *brevitarsis*	3279	828	254	4361
*C*. *bundyensis*	3074	1254	64	4392
*C*. *bunrooensis*	232	387	478	1097
*C*. *dycei*	92	1112	8889	10093
*C*. *fulbrighti*	923	2		925
*C*. *loughnani*		10	3	13
*C*. *marginalis*		28	22	50
*C*. *marksi*	777	15470	42685	58932
*C*. *moreensis*	1			1
*C*. *nattaiensis*	295	148	1291	1734
*C*. (Ornatus Gp sp) **#**8		133	11	144
*C*. *parvimaculatus*		1	1	2
*C*. *rabauli*	21	17	1	39
*C*. *shermani*		2	3	5
*C*. *sigmoidus*	27	1		28
*C*. *victoriae*	10677	3862	171	14710
*C*. *williwilli*	15	22	1	38
*C*. *zentae*		35	1	36
Total	23823	41522	87284	152629
**Species diversity**	**13**	**18**	**16**	
**Diversity index(H)**	**1.59**	**1.33**	**1.08**	
**Evenness index (E)**	**0.62**	**0.46**	**0.39**	

^a^Numbers in brackets represent number of locations grouped in each subregion.

## Discussion

Based on monitoring of *Culicoides*-borne viruses between 1990 and 2018, nineteen *Culicoides* species identified from the New England region of NSW, Australia are reported in this study. With trapped numbers in excess of 56,000 each, the most abundant species were *C*. *marksi* (38.6%) and *C*. *austropalpalis (*36.7%), with eight of the species caught making up 99.2% of the total catch. Forty-nine *Culicoides* species have previously been reported in NSW [10], of which 39% were detected in this study. Seasonal abundance as measured by mean counts per trapping event for the eight most abundant species, revealed a non-significant trend towards increase in abundance of *C*. *marksi* and *C*. *austropalpalis* in summer. Within the New England region, the abundance of *C*. *marksi*, *C*. *austropalpalis* and *C*. *dycei* declined with increasing altitude from the plains to the slopes and tablelands while *C*. *victoriae* exhibited a non-significant trend (P = 0.08) in the opposite direction. The biting midge, *C*. (Ornatus Gp sp) **#**8, was detected in the region from 2015/16 onwards with its distribution limited to the plains and slopes. Dyce et al. [10] has previously mentioned the presence of this species in NSW. *Culicoides brevitarsis*, a major vector of livestock diseases comprised 2.9% of the total catch and was detected in 12 of the 13 locations in the study.

The overall increase in species abundance and detection following introduction of the green LED trap since 2005/2006 (Change 1&2; S1 Table) was likely due to the better performance of the trap as was reported previously [28]. The response of *C*. *marksi*, *C*. *austropalpalis*, *C*. *dycei*, *C*. *victoriae*, *C*. *bunrooensis* and *C*. *bundyensis* to the green LED lamp design improvement was significant and consistent with previous reports [28] except for *C*. *nattaiensis* which exhibited reduced catches with the improved trap type. Though an increase in mean counts with green LED was observed for *C*. *brevitarsis*, the response was not significant unlike previous studies [28, 30, 40]. This may be due to the comparatively low catch rate of this species in the present study. The change in trap type was associated with detection of new *Culicoides* species, *C*. *loughnani*, *C*. (Ornatus Gp sp) **#**8, and *C*. *moreensis* which could be due to better detection with the improved trap type or potential blow-ins (insects arriving on the wind). The overall increase in catches for eight of most abundant species since 2016/17 may be associated with improvements to the green LED lamp design but since there was no simultaneous deployment of trap types, the increase cannot be definitively attributed to the efficiency of the trap types used.

The trend towards higher counts of *C*. *marksi* and *C*. *austropalpalis* in summer was consistent with a decline in abundance of *C*. *marksi* and *C*. *austropalpalis* with decreasing temperature in the Hunter Valley of NSW [43]. Summer is the season with the highest rainfall in the region and this may have created favourable breeding conditions for the aquatic larvae of these species [13]. In NSW, *C*. *brevitarsis* dispersal from over-wintering foci on the mid-northern/northern coastal plain was reported to occur in spring and summer [44, 45] through to near the end of autumn [46], with present findings in agreement with these observations. However, a study from the coastal plains east of the study region found that the peak season for *C*. *brevitarsis* is summer [47]. The continuous detection of *C*. *brevitarsis* in the present study, with the availability of hosts, ideal temperatures of 20-25°C during the longer day light periods in spring and summer [43] may indicate establishment of the species on the tablelands as opposed to presence based on seasonal waves of movement [46]. This proposition requires further investigation as establishment could potentially be masked by *C*. *brevitarsis* dispersal from coast [48]. The arithmetically highest catch rate of *C*. *brevitarsis* was in autumn and this may be of significance in the region as a model showed proportion of potentially infected/infective *C*. *brevitarsis* dispersing from the coast increases with length of the season [1, 37, 46, 49]. Most predictions related to the seasonal dispersal of *Culicoides* are based on *C*. *brevitarsis* [44, 48] and studies on the habitat requirements of the other species could provide better understanding of their seasonal dispersal in the region.

The marked preference of *C*. *dycei*, *C*. *marksi* and *C*. *austropalpalis* for the lower altitude plains and slopes is likely due to less severe winters or a combination of factors such as availability of hosts, larval habitat, climate tolerance and longevity [13, 27, 29, 30]. In support of a preference for warmer temperatures, a study on simultaneous detection of *C*. *marksi* and *C*. *austropalpalis* in the NSW and Northern Territory (NT) with green LED traps yielded more counts in warmer NT [28]. *Culicoides victoriae* exhibited the reverse trend suggestive of a tolerance of a wider temperature range and consistent with this, it has been reported in all eastern states [10]. A model [46, 48] on the dispersal of *C*. *brevitarsis* through coastal valleys to central and northwest parts of NSW showed a delay in detection of the species determined by distance from the coast and altitude. The results of the present study are consistent with this prediction as in most cases the first detection on the tablelands was recorded in mid-summer. Trapping sites on the tablelands are situated on the top of the Great Dividing Range (GDR) where easterly winds from the coast during autumn might have sped up the rate of dispersal and caused relatively higher mean counts of *C*. *brevitarsis*. Moreover, the detection of *C*. *brevitarsis* on the New England plains provided evidence of its rare incursion and crossing the GDR [46], however as noted above, establishment of the species in the New England region cannot be ruled out. Further investigations on the role of bioclimatic variables determining the dispersal of most abundant species collected in the present study is recommended for future modelling [29, 50].

Despite the greater abundance and richness of *Culicoides* species, the plain and slope subregions had lower diversity and evenness indices. This is due to the dominance of *C*. *marksi* and *C*. *austropalpalis* in these subregions. The highest species richness [18] was observed on the slopes possibly be due to water run-off from the tablelands creating favourable breeding ecosystems for the immature stages of *C*. *austropalpalis* and *C*. *marksi* [51] though a slope of more than 5° has been reported detrimental [52]. There is also an intermediate zone between the plains and tablelands with a more diverse array of potential habitats, including those suitable for species that prefer either the plains or tableland habitats. The relatively small number of *C*. *brevitarsis* on the slopes and plains [46] relative to the tablelands may be due increasing dryness and reduced availability of larval breeding habitat as the area is largely used for crop production. This would be consistent with the suggestion that the distribution of *C*. *brevitarsis* to the west of NSW is limited by the availability of moisture [53]. However, the higher prevalence on the tablelands may also simply reflect closer proximity to coastal breeding grounds with wind borne ingress onto the tablelands.

## Conclusion

This study provides the first comprehensive list of *Culicoides* species found in the New England region of NSW, Australia between 1990 and 2018. Nineteen species were identified with eight of the most abundant species making up over 99% of the total trap catch. *Culicoides marksi* and *C*. *austropalpalis* were the most abundant and widespread species. There was a significant increase in the trapping rate of *C*. *marksi*, *C*. *austropalpalis*, *C*. *dycei*, *C*. *victoriae*, *C*. *bunrooensis* and *C*. *bundyensis* with the change to green LED lamps although this was not the case for *C*. *nattaiensis*. A trend with higher abundance was observed during the warmer seasons and at lower altitudes for *C*. *marksi*, *C*. *austropalpalis* and *C*. *dycei* indicating dispersal of these species in the New England primarily dependent on temperature and rainfall. However, no marked difference in abundance of *C*. *brevitarsis* across seasons and subregions was observed and it is unclear to what extent the species is established in the region. Further research on bioclimatic factors influencing temporal and spatial dispersal of the different *Culicoides* species is recommended which will assist with modelling their dispersal and the potential dispersal of arboviruses that may affect livestock and wildlife species in the future.

## Supporting information

S1 Table Abundances of *Culicoides* species trapped in the New England region of NSW, Australia across the twenty-six trapping years. (DOCX)
