# Supplementary material for: Spatial and temporal distribution of Culicoides species in the New England region of New South Wales, Australia between 1990 and 2018
Source: PLoS One. 2021 Apr 5;16(4):e0249468. doi: 10.1371/journal.pone.0249468 (PMC8021189; doi:10.1371/journal.pone.0249468)
Supplement: S1 Table — (DOCX) [file pone.0249468.s001.docx]

**S1 Table. Abundances of *Culicoides* species trapped in the New England region of NSW, Australia across the twenty-six trapping years.**

| Trapping Years | *C. austropalpalis* | *C. brevitarsis* | *C. bundyensis* | *C. bunrooensis* | *C. dycei* | *C. fulbrighti* | *C. loughnani* | *C. marginalis* | *C. marksi* | *C. moreenensis* | *C. nattaiensis* | *C.* (*Ornatus* Gp sp) **#***8* | *C. parvimaculatus* | *C. rabauli* | *C. shermani* | *C. sigmoidus* | *C. victoriae* | *C. williwilli* | *C. zentae* |
| --- | --- | --- | --- | --- | --- | --- | --- | --- | --- | --- | --- | --- | --- | --- | --- | --- | --- | --- | --- |
| 1990/91 | 1095 | 5 | 199 | 32 | 81 |  |  | 1 | 928 |  | 46 |  |  |  |  |  | 471 | 2 | 25 |
| 1991/92 | 929 | 8 | 121 | 68 | 109 |  |  |  | 455 |  | 42 |  | 2 |  |  |  | 765 | 2 | 11 |
| 1994/95 | 5 |  |  | 4 | 3 |  |  |  | 5 |  |  |  |  |  |  |  | 2 |  |  |
| 1995/96 | 2531 |  |  | 30 | 481 |  |  |  | 766 |  | 259 |  |  |  | 2 |  | 5 |  |  |
| 1996/97 | 112 | 7 | 31 | 2 | 48 |  |  |  | 37 |  | 61 |  |  |  |  |  | 34 |  |  |
| 1997/98 | 598 | 233 | 109 | 4 | 116 |  |  |  | 2140 |  | 116 |  |  |  |  |  | 267 |  |  |
| 1998/99 | 280 |  | 60 | 11 | 85 |  |  |  | 93 |  | 167 |  |  |  |  |  | 121 |  |  |
| 1999/00 | 222 | 29 | 113 | 15 | 40 | 18 |  |  | 70 |  | 70 |  |  |  |  | 1 | 232 |  |  |
| 2000/01 | 522 | 34 | 112 | 53 | 145 | 19 |  |  | 1615 |  | 134 |  |  |  |  |  | 166 |  |  |
| 2001/02 | 1460 | 41 | 75 | 37 | 1102 |  |  |  | 1469 |  | 50 |  |  |  | 1 | 1 | 126 |  |  |
| 2002/03 | 2365 | 60 | 22 | 8 | 1291 |  |  |  | 1283 |  | 19 |  |  |  |  |  | 293 |  |  |
| 2003/04 | 3132 | 19 | 37 | 47 | 1051 | 8 |  |  | 8275 |  | 353 |  |  |  | 2 | 2 | 309 |  |  |
| 2004/05 | 126 |  | 6 |  |  |  |  |  | 11 |  | 3 |  |  | 2 |  |  | 128 |  |  |
| 2005/06 | 471 | 170 | 29 | 5 | 124 | 3 |  |  | 1808 |  | 10 |  |  | 1 |  |  | 236 |  |  |
| 2006/07 | 957 | 146 | 294 | 11 | 593 | 30 |  |  | 673 |  | 15 |  |  |  |  | 3 | 894 |  |  |
| 2007/08 | 656 | 7 | 95 | 23 | 134 | 22 |  |  | 53 |  | 2 |  |  | 1 |  |  | 171 |  |  |
| 2008/09 | 1144 | 18 | 464 | 9 | 325 | 67 |  |  | 311 |  | 21 |  |  |  |  | 4 | 830 |  |  |
| 2009/10 | 7601 | 191 | 185 | 46 | 763 |  |  |  | 7407 |  | 10 |  |  |  |  | 3 | 773 |  |  |
| 2010/11 | 206 | 25 | 239 | 17 | 24 | 19 | 1 |  | 97 |  | 32 |  |  | 1 |  |  | 514 |  |  |
| 2011/12 | 1519 | 181 | 235 | 85 | 172 | 42 | 1 | 2 | 1456 |  | 36 |  |  | 1 |  |  | 848 |  |  |
| 2012/13 | 1115 | 183 | 67 | 48 | 443 | 18 |  |  | 4481 |  | 5 |  |  |  |  | 1 | 343 | 2 |  |
| 2013/14 | 3397 | 1723 | 11 | 52 | 123 | 1 |  | 1 | 1148 |  | 1 |  |  |  |  | 7 | 323 |  |  |
| 2014/15 | 1416 | 818 | 176 | 19 | 243 | 5 | 1 |  | 1404 |  | 11 |  |  | 2 |  | 1 | 453 | 11 |  |
| 2015/16 | 4158 | 18 | 85 | 15 | 105 | 32 | 1 | 2 | 1054 |  | 11 | 1 |  | 7 |  | 2 | 234 | 2 |  |
| 2016/17 | 7371 | 39 | 1132 | 342 | 670 | 182 | 1 | 18 | 5799 |  | 242 | 4 |  | 15 |  | 2 | 3380 | 3 |  |
| 2017/18 | 12641 | 406 | 495 | 114 | 1822 | 459 | 8 | 26 | 16094 | 1 | 18 | 139 |  | 9 |  | 1 | 2792 | 16 |  |
